# Effects of High-Intensity Training on Anaerobic and Aerobic Contributions to Total Energy Release During Repeated Supramaximal Exercise in Obese Adults

**DOI:** 10.1186/s40798-015-0035-7

**Published:** 2015-10-20

**Authors:** Georges Jabbour, Horia-Daniel Iancu, Anne Paulin

**Affiliations:** School of Kinesiology and Leisure, Faculty of Health Sciences and Community Services, Université de Moncton, Moncton, NB E1A 3E9 Canada

**Keywords:** High-intensity training, Aerobic, Anaerobic, Energy contribution

## Abstract

**Background:**

Studying relative anaerobic and aerobic metabolism contributions to total energy release during exercise may be valuable in understanding exercise energetic demands and the energetic adaptations that occur in response to acute or chronic exercise in obese adults. The aim of the present study is to evaluate the effects of 6 weeks of high-intensity training (HIT) on relative anaerobic and aerobic contributions to total energy release and on peak power output during repeated supramaximal cycling exercises (SCE) in obese adults.

**Methods:**

Twenty-four obese adults (body mass index = ± 33 kg.m^−2^) were randomized into a control group (*n* = 12) and an HIT group (*n* = 12). Accumulated oxygen deficits (ml.min^−1^) and anaerobic and aerobic contributions (%) were measured in all groups before and after training via repeated SCE. In addition, the peak power output performed during SCE was determined using the force-velocity test.

**Results:**

Before HIT, anaerobic contributions to repeated SCE did not differ between the groups and decreased significantly during the third and fourth repetitions. After HIT, anaerobic contributions increased significantly in the HIT group (+11 %, *p* < 0.01) and were significantly higher than those of the control group (*p* < 0.01). Moreover, the peak power obtained during SCE increased significantly in the HIT group (+110 W.kg^−1^, *p* < 0.01) and correlated positively with increases in anaerobic contributions (*r* = 0.9, *p* < 0.01).

**Conclusions:**

In obese adults, HIT increased anaerobic contributions to energy release which were associated with peak power enhancement in response to repeated SCE. Consequently, HIT may be an appropriate approach for improving energy contributions and muscle power among obese adults.

## Key Points

- Anaerobic contributions to energy release increased significantly in the trained group following HIT. These higher anaerobic contributions were accompanied by both greater AOD deficits and larger decreases in oxygen uptake during SCE repetitions.
- HIT may be an appropriate approach for enhancing the level of substrate phosphorylation from PCr hydrolysis and glycolysis during repeated supramaximal exercise bouts, which may be partially related to enhancements in the muscle efficacy of obese adults.
- In obese adults, studying relative anaerobic and aerobic metabolism contributions to total energy release during exercise may be valuable in understanding exercise energetic demands and the energetic adaptations that occur in response to acute or chronic exercise.

## Background

Several studies have recently focused on determining energetic system contributions in several sports practices, with the objective of understanding the energetic demand related to these events in order to develop improved training recommendations [1, 2]. In the context of obesity, exercise energy metabolism is impaired [3, 4]. Aerobic energy release during peak exercise is reduced by excessive fat mass, which imposes an unfavorable burden on cardiac function and on oxygen uptake by working muscles [5]. Evaluation of aerobic energy release has become part of routine clinical assessments of this population, as the degree of its reduction is a strong predictor of an individual’s health [6]. By contrast, anaerobic metabolic pathways have received less attention. Studies involving obese individuals are more focused on evaluating anaerobic aptitude (e.g., maximal power output). These studies describe lower values of peak power output in obese subjects compared with normal-weight subjects [7, 8]. This reduced parameter has been linked to excessive fat mass, which may reduce motor-unit activation and the ratio of muscle strength to units of muscle mass [8]. However, no data are available regarding anaerobic energetic yield during exercise. Therefore, studying energetic muscle interactions for a given exercise may be the best approach to describing the true metabolic energy of the muscles of obese individuals and understanding the metabolic adaptations that occur in response to an exercise intervention in obese individuals.

Regarding intervention strategies and their impact on metabolic profiles, high-intensity training, such as supramaximal exercise, is gaining popularity in the context of obesity management. Based on data provided by post-intervention metabolic measures, this model of exercise appears to be a valuable approach for managing obesity [9]. Indeed, Whyte et al. [10] demonstrated that very high-intensity sprint interval training [2 weeks, comprising 6 sessions of 4 to 6 repeats of 30-s Wingate anaerobic sprints on an electromagnetically braked cycle ergometer, with a 4.5-min recovery period between each repetition] improved several metabolic risk factors: 1) an increase in resting fat oxidation rate in the fasted state and 2) a decrease in resting carbohydrate oxidation in the fasted state compared with baseline. This high-intensity training also decreased waist and hip circumferences in overweight and obese sedentary men and illustrated the potential of this alternative exercise model to improve lipid utilization in individuals with excess body weight. However, no information describing true metabolic adaptations during exercise is available. More importantly, a simultaneous evaluation of the maximal activation of both energetic systems (e.g., anaerobic vs. aerobic) may allow us to better understand anaerobic/aerobic interactions during exercise and provide information regarding the factor(s) that limit performance in obese individuals, as well as for other populations (normal-weight individuals, athletes).

Accumulated oxygen deficit (AOD) is a descriptor of the maximal amount of energy obtained from anaerobic metabolism [11] and makes it possible to directly measure maximal anaerobic energy release during supramaximal effort. This concept makes it possible to simultaneously evaluate the maximal activation of both anaerobic and aerobic metabolic systems and obtain a complete and coherent description of exercise energy demands in obese individuals. Based on the Oxygen consumption (VO_2_) predicted from the individual VO_2_-intensity relationship determined from the graded exercise test, the AOD can be determined during supramaximal exercise protocol [12]. It has been admitted that AOD measures are generally accepted as the criterion measure of anaerobic energy expenditure as a result of flaws in the use of blood lactate concentration as a quantitative tool for the measurement of anaerobic energy supply [13].

The aim of the present study is to evaluate the relative anaerobic and aerobic contributions to total energy release during repeated bouts of supramaximal cycling exercise in obese adults undergoing 6 weeks of supramaximal exercise training. Therefore, the examination of muscle metabolism and power output profiles in response to repeated bouts of cycling sprinting with a short recovery interval may enable us to better understand the regulation of these metabolic pathways.

## Methods

### Experimental Subjects

Twenty-four young adults (13 women and 11 men) were recruited from the Moncton campus of the University of Moncton. To do so, we invited by posting announcement to voluntarily participate students of the University of Moncton who applied the inclusion criteria. The study protocol was approved by the University’s Human Research Ethics Committee (UHRC), and all procedures followed were in accordance with the Helsinki Declaration of 1975, as revised in 2008. Informed consent was obtained from all patients for being included in the study. In addition to being obese, the inclusion criteria for participation were as follows: participants had to be physically active fewer than 60 min.week^−1^ as assessed by the International Physical Activity Questionnaire [14] and have no history of metabolic, cardiovascular, or chronic health problems, no history of drug consumption before the study, and no history of smoking. Before entering our protocol, each of the participants was thoroughly familiarized with all testing equipment and procedures. Indeed, each subject cycled for an extended period of time on the same cycle ergometer used throughout the study. Additionally, each participant was asked to determine the height of seat at which they are able to pedal comfortably. Unfortunately, we do not have any objective positioning (knee angle, hip angle, etc.) and this may be considered as limitation of the study. But, it is important to note that the position of each participant, e.g., the seat height, was the same throughout the study.

The protocol then began with three sessions of preliminary testing in order to determine certain key variables. The testing was conducted on three different days (D1, D2, and D3) after an overnight fast. Each day was separated by a minimum of 48 h, and all subjects were asked to avoid physical activity for 48 h prior to each session. In addition, subjects were asked to be well hydrated before each test. The same protocol that has been previously developed by our laboratory [15] seems suitable to delay any early fatigue or discomfort among participants which were all sedentary.

Following the determination of body composition, obese participants (BMI >30 kg/m^2^) were selected based on the Canadian guidelines for body weight classification in adults [16] and were separated in the following two groups: a control group (without any intervention; *n* = 12) and a training group (*n* = 12). Fat-free mass was calculated by subtracting fat mass from body mass.

### Anthropometric Measurements

Body mass was measured to the nearest 0.1 kg with the subject in light clothing, without shoes, using an electronic scale (Kern, MFB 150 K100). Height was determined to the nearest 0.5 cm with a measuring tape fixed to the wall. Body mass index (BMI) was calculated as the ratio of mass (kg) to height^2^ (m^2^). Body fat percentage was estimated using a bioimpedance machine (Vacumed, Bodystat1500).

### Physiology Assessment

On day 1 (D1), subjects performed a maximal test on an upright cycle ergometer (Monark ergomedic 839E electronic test cycle, USA) to determine their peak oxygen consumption (VO_2peak_). Before beginning the test, adults remained seated for 5 min on the bicycle ergometer in the same position used in subsequent exercise. Resting oxygen consumption was measured based on the mean oxygen consumption of the last 30 s of minutes 3, 4, and 5. No proper warm-up was performed. The test started at an initial power of 25 W and was progressively increased by 25 W every 2 min until exhaustion. In the present study, all participants reach exhaustion at 125 W. In fact, those who could not achieve a power output greater than or equal to 125 W were excluded.

A breath-by-breath automated metabolic system (CPX, Medical Graphics, St. Paul, Minnesota, USA) was used to determine the VO_2peak_ of each participant. Calibration prior to each test was performed with standard gases of known oxygen and carbon dioxide concentration for gas composition and a calibration syringe for volume. The data were averaged on a 30-s interval, and oxygen uptake and respiratory exchange ratio, which is the ratio of carbon dioxide produced to oxygen consumed, were obtained.

Peak oxygen consumption was achieved when a subject fulfilled at least three of the following criteria: a plateau in VO_2_ in spite of an increase in exercise intensity, a respiratory exchange ratio greater than 1.1, a maximal HR above 90 % of the predicted maximal theoretical HR (220—age in years) or apparent exhaustion [17].

On day 2 (D2), we measured steady-state VO_2 uptake_ at a constant submaximal power (below the VO_2peak_). After a 3-min resting baseline period, subjects started pedaling at the established power, which was maintained for 10 min at a pedaling rate of 60 revolutions/min (rpm). The 10-min exercise bouts were completed 6 times for each subject at powers of 30, 40, 50, 60, 70, and 80 % of their individual VO_2peak_ power and were separated by a 5-min resting period, and the measurements for each subject were plotted separately and visually checked for linearity as described elsewhere [18]. This demonstrates that our measured VO_2 uptake_ for a 10-min exercise was a steady-state value equaling VO_2 demand_ (total rate of energy release). We therefore conclude that VO_2 demand_ increased linearly with power for all subjects in the examined range. This analysis allowed for the calculation of the accumulated oxygen deficit (AOD) (measured in ml O_2_ equivalents per kg) for each cycling interval by calculating the difference between VO_2 demand_ for the respective power (from extrapolation of the calculated relationship) and VO_2 uptake_. The linear relationship between steady-state VO_2_ values and cycling power was extrapolated and used to estimate energy demand during supramaximal cycling exercise (SCE) [19].

On day 3 (D3), following 10 min of warm-up, subjects performed a force-velocity test on a cycle ergometer using a technique adapted from the study performed by Vandewalle et al. [20]. This test consists of a succession of supramaximal bouts of approximately 6 s, with exercise loads increasing by 1 kg after each bout until the subject is unable to perform the test. A period of passive recovery (5 min) was allowed between successive bouts. The peak velocity for each bout was recorded, and the power output was calculated by multiplying the load and speed. The optimal load corresponded to the load at which maximal power (PO_max_) was achieved. This load was then used for the training protocol that followed. The force-velocity test was also performed every 2 weeks to adjust the individual power level of SCE. These 3 days of testing were completed before high-intensity training (HIT) (visit 1), and also at the end of training (visit 2).

### Training Session

Once participants completed preliminary testing, they were instructed to complete a total of 18 training sessions (three sessions per week for 6 weeks). Each of the prescribed sessions began with a 5-min warm-up of continuous cycling at moderate intensity (40 % of their individual VO_2peak_ power), followed by 6 repetitions of SCE intervals with 2 min of passive recovery between each repetition. Each SCE repetition lasted 6 s, and participants were asked to pedal at maximal velocity against the resistance determined during D3. The repeat sprint cycling test was conducted under the supervision of a member of the research team, and velocities (in RPM) were recorded for each second of the bout in order to ensure that said velocities were constant. Based on the linear regression and the individual VO_2max_, the workload approximately corresponded to ~350 % of VO_2max_ [12].

In fact, we have chosen in the present study the HIT for many reasons. Firstly, very brief high-intensity repeated exercise comprised of 6- to 10-s sprints induces substantial improvements in both performance and health-related outcomes [21]. For example, 2 to 15 weeks of this type of training results in significant increases in anaerobic capacity, ranging between 5 and 28 % in untrained males [21]. Moreover, it represents a time-efficient approach to obtain health benefits from exercise, as sessions involve a total of only 2–3 min of high-intensity exercise and typically last for less than 15 min [22, 23].

Training sessions were conducted under the supervision of a member of the research team, and velocities (in RPM) were recorded for each second of the bout in order to ensure that said velocities were constant. Additional testing was also completed during the first prescribed training session, as well as during the training session at the end of the sixth week. Indeed, during the first and the final sessions, participants were asked to perform one of their regular training sessions while being analyzed by our breath-by-breath automated metabolic system during only four repetitions. This was done in order to determine the contribution of each energy system. In this study, results are given only from four supramaximal cycling exercise bout given that some of participants felt discomfort by wearing mask destined for gas analysis. Then, the mask was removed to allow participants to complete the entire session bouts. Lactate concentrations were obtained at rest for all experimental subjects, and immediately following the fourth repetition, via capillary blood samples using the LactatePro analyzer. This technique was previously utilized in our laboratory to monitor athletes. In the present study, lactate concentrations served only to complete the profiles of the control and training groups, before and after HIT.

After completing 6 weeks of training, participants were asked to return for a final day of testing (visit 2), during which the procedures of D1, D2, and D3 were repeated, and post-training data was collected.

### Calculation of Relative Energy Expenditure: Supramaximal Cycling Exercise Bout

The VO_2 demand_ values of SCE were estimated individually by extrapolating the linear relationship between the power and the VO_2 demand_ values established during the constant submaximal power exercises. The accumulated VO_2 uptake_ and the accumulated VO_2 demand_ were taken as the VO_2 uptake_ and the VO_2 demand_ integrated over the entire supramaximal cycling exercise bout [18]. The AOD was equal to the accumulated VO_2 demand_ minus the accumulated VO_2 uptake_. This allowed for a measurement of anaerobic (AOD) and aerobic (VO_2_) energy contributions throughout the four SCE repetitions [24].

### Statistical Analyses

After testing for normality (Kolmogorov–Smirnov test), statistical comparisons were made between the control group and the training group on two separate occasions (before and after training). Two-way repeated measures ANOVA was used to determine whether significant changes in energy system contributions emerged between the two groups, and if energy system contributions differed between the two groups. Furthermore, Bonferroni’s post hoc test was performed. Pearson correlations were used to assess the relationship among changes in muscle power, aerobic capacity, and energy contribution modification. A value of *p* < 0.05 was statistically significant. Analyses were performed using IBM SPSS Statistics 19 software.

## Results

Height, body mass, body mass index, fat mass, and fat-free mass were similar across the two groups during visit 1 and visit 2 (Table 1). Resting heart rates were lower for the trained group during visit 2 compared with visit 1 (*p* < 0.01) and were also lower than those of the control group during visit 2 (*p* < 0.01). VO_2max_ values assessed after 6 weeks of HIT training did not differ from those of visit 1 and visit 2. The maximal power output obtained during the force-velocity test increased significantly in the training group compared with visit 1 and was significantly higher than the corresponding values of the control group for visit 1 and visit 2 (Table 1).

**Table 1:** Age, anthropometric, aerobic and anaerobic fitness parameters of young obese adults at visit 1 and visit 2

	Visit 1		Visit 2		Δ Visit 2 vs. visit 1 for control group	Δ Visit 2 vs. visit 1 for trained group
	Control	Trained	Control	Trained		
	(*n* = 12)	(*n* = 12)	(*n* = 12)	(*n* = 12)		
	(*w* = 7, *m* = 5)	(*w* = 6, *m* = 6)	(*w* = 7, *m* = 5)	(*w* = 6, *m* = 6)		
Age and anthropometrics						
Age (year)	23.1 (3.3)	22.5 (2.3)	23.3 (2.3)	22.7 (2.2)	0.2	0.2
Height (cm)	1.71 (0.11)	1.74 (0.09)	1.71 (0.11)	1.74 (0.09)	–	–
Body mass (kg)	99.5 (24.1)	101.1 (21.1)	100.5 (21.1)	99.9 (9.1)	1	−1.1
BMI (kg.m^−2^)	33.3 (4.8)	33.2 (2.8)	33.7 (3.8)	33.1 (3.7)	0.4	−0.1
FM (%)	42.3 (9.4)	42.8 (7.4)	44.3 (9.4)	42.1 (7.1)	2	−0.6
FFM (kg)	51.2 (10.1)	50.2 (9.1)	50.2 (9.1)	50.8 (5.7)	−1	0.5
Aerobic and anaerobic fitness indicators						
HR peak (beats.min^−1^)	196 (12)	196 (09)	197 (16)	198 (12)	1	−2
RERpeak	1.1 (0.1)	1.1 (0.3)	1.1 (0.3)	1.1 (0.2)	–	–
P_VO2max_ (W)	125 (0.1)	125 (0.1)	125 (0.1)	125 (0.1)	–	–
VO_2max_ (ml.min^−1^.kg^−1^)	23.4 (8.4)	22.6 (6.4)	22.2 (7.4)	23.1 (6.1)	−1.2	0.5
PO_max_ (W)	470 (30)	465 (35)	465 (25)	570 (40)*^,^**	−5	105

Values are expressed as the mean (standard deviation)

*w* woman, *m* men, *BMI* body mass index, *FM* fat mass, *FFM* fat-free mass, *HR* heart rate, *RER* respiratory exchange ratio, *P*_*VO2max*_ maximal power output obtained during the maximal test, *VO*_*2max*_ maximal oxygen consumption, *PO*_*max*_ maximal power output developed during the force-velocity test, *Δ* variation between parameters assessed at visit 1 and visit 2

*Significant difference between groups (*p* < 0.01); **significant difference from visit 1 (*p* < 0.01)

During all SCE bouts, heart rate values did not differ among the groups (Table 2). For the trained group, the power output exhibited during SCE was significantly higher compared with that observed during visit 1 (*p* < .0.1) and was significantly different compared with that of the control group during visit 1 (*p* < 0.01) and visit 2 (*p* < 0.01), and compared with the fourth repetitions. VO_2__uptake_ did not differ statistically across visits for the control group. However, following HIT, the VO_2__uptake_ values determined during the first, second, third, and fourth repetitions decreased significantly in the trained group (*p* < 0.01) and were significantly lower than the values of the control group obtained during visit 1 (*p* < 0.01) and visit 2 (*p* < 0.01) (Table 2). For both the control group and the trained group, VO_2__uptake_ increased significantly during the third and fourth repetitions compared with values obtained during the first repetition, before HIT (*p* < 0.01) (Table 2). By contrast, the AOD decreased for the trained group before HIT, and for the control group during the third and foruth repetitions (*p* < 0.01). Following HIT, the trained group demonstrated significantly higher AOD values compared with those obtained before HIT (*p* < 0.01), and compared with the control group (*p* < 0.01). Additionally, the VO_2__uptake_, the AOD, the aerobic, and the aerobic contributions determined following HIT remained constant during all repetition bouts (Table 2).

**Table 2:** Calculated and measured variables during the supramaximal cycling exercise (SCE)

	Visit 1		Visit 2		Δ Visit 2 vs. visit 1 for control group	Δ Visit 2 vs. visit 1 for trained group
	Control	Trained	Control	Trained		
	(*n* = 12)	(*n* = 12)	(*n* = 12)	(*n* = 12)		
	(*w* = 7, *m* = 5)	(*w* = 6, *m* = 6)	(*w* = 7, *m* = 5)	(*w* = 6, *m* = 6)		
	First repetition					
HR (beats.min^−1^)	112.1 (2.8)	117.3 (4.1)	117.2 (3.1)	113.4 (4.1)	5.1	−4.3
PO (W)	470 (30)	465 (35)	470 (25)	600 (40)*^,^**	0	135
VO_2 uptake_ (ml.kg^−1^.min^−1^)	14.1 (3.7)	13.5 (3.2)	13.6 (3.7)	6.1 (3.1)*^,^**	−0.5	−7.4
AOD (ml.kg^−1^)	72.4 (1.4)	70.4 (1.4)	70.1 (3.4)	104.5 (3.4)*^,^**	−2.3	34.1
Aerobic contribution (%)	17	17	16	6*^,^**	−1	−11
Anaerobic contribution (%)	83	83	84	94*^,^**	1	11
	Second repetition					
HR (beats.min^−1^)	113.1 (2.9)	114.3 (4.2)	114.2 (2.1)	114.4 (3.1)	1.1	0.1
PO (W)	470 (30)	465 (35)	470 (25)	600 (40)*^,^**	0	135
VO_2 uptake_ (ml.kg^−1^.min^−1^)	16.1 (3.7)	15.5 (3.2)	15.6 (3.7)	8.1 (3.1)*^,^**	0.5	−7.4
AOD (ml.kg^−1^)	70.4 (0.5)	69.1 (0.9)	68.1 (1.1)	103.1 (0.5)*^,^**	−2.4	34
Aerobic contribution (%)	19	18	19	7*^,^**	0	−11
Anaerobic contribution (%)	81	82	81	93*^,^**	0	11
	Third repetition					
HR (beats.min^−1^)	117.9 (1.8)	119.3 (3.1)	119.2 (3.1)	116.4 (4.1)	1.3	−2.9
PO (W)	470 (30)	465 (35)	470 (25)	600 (40)*^,^**	0	135
VO_2 uptake_ (ml.kg^−1^.min^−1^)	19.1 (3.7)***	19.5 (3.2)***	18.6 (3.7)***	7.1 (3.1)*^,^**	−0.5	−12.4
AOD (ml.kg^−1^)	67.4 (1.2)***	65.2 (3.1)***	64.8 (2.1)***	103.9 (5.3)*^,^**	−2.6	38.7
Aerobic contribution (%)	22.6***	22.4***	22.4***	7*^,^**	−0.2	−15.4
Anaerobic contribution (%)	77.4***	77.6***	77.6***	93*^,^**	0.2	15.4
	Fourth repetition					
HR (beats.min^−1^)	118.9 (0.8)	117.3 (2.1)	118.2 (3.1)	117.4 (3.1)	−0.6	0.1
PO (W)	470 (30)	465 (35)	470 (25)	600 (40)*^,^**	0	135
VO_2 uptake_ (ml.kg^−1^.min^−1^)	19.3 (3.1)***	19.6 (1.2)***	18.4 (2.1)***	6.9 (2.1)*^,^**	−0.9	−12.7
AOD (ml.kg^−1^)	67.1 (1.3)***	65.1 (1.1)***	65.1 (2.1)***	104.1 (1.5)*^,^**	−2	39
Aerobic contribution (%)	22.5***	22.5***	21.9***	6.4*^,^**	−0.6	−16.1
Anaerobic contribution (%)	77.5***	77.5***	78.1***	93.6*^,^**	0.6	16.1
Lactate rest (mmol.L^−1^)	1.9 (0.5)	2.1 (0.5)	2.1 (0.3)	2.1 (0.5)	0.2	0
Lactate fourth repetition (mmol.L^−1^)	7.2 (2.5)	7.3 (2.5)	7.1 (2.5)	13.4 (3.1)*^,^**	−0.1	6.1

Values are expressed as the mean (standard deviation)

*w* woman, *m* men, *HR* heart rate, *PO* maximal power output developed during the SCE, *VO*_*2 uptake*_ oxygen uptake during repetition, *AOD* accumulated oxygen, *Δ* variation between parameters assessed at visit 1 and visit 2

*Significant difference between groups (*p* < 0.01); **significant difference from visit 1 (*p* < 0.01); ***significant difference from the first repetition (*p* < 0.01)

Anaerobic energy contributions (calculated using the AOD method) increased significantly for the training group following HIT (*p* < 0.01) and were also significantly higher during SCE during the first, second, third, and fourth repetitions compared with values obtained for the control group during visit 1 (*p* < 0.01) and visit 2 (*p* < 0.01). During the third and the fourth repetitions, the control group and the trained group demonstrated significant decreases in anaerobic contributions compared with values obtained during the first repetition, decreases that were accompanied by increases in the values of the aerobic contributions, before HIT (Table 2).

Lactate concentrations measured at rest did not differ between the groups during visit 1 and visit 2. Following HIT, lactate concentrations increased significantly in the trained group compared with visit 1 (~6 mmol.kg ^−1^, *p* < 0.01) and were significantly higher than those observed for in the control group during visit 1 and visit 2 (*p* < 0.01).

In this study, the increased power output levels observed for the trained group following HIT and during the SCE bouts correlated positively with anaerobic contribution increases (*r* = 0.9, *p* < 0.01, respectively). However, no relationships were observed between anaerobic contribution increases and VO_2max_ (*r* = 0.08, *p* = 0.7).

## Discussion

To the best of our knowledge, this study is the first to examine the effects of HIT on relative anaerobic and aerobic contributions to total energy release in obese adults during repeated bouts of supramaximal cycling exercise. The primary finding of this study was that relative anaerobic and aerobic contributions to total energy release during repeated bouts of SCE differed in untrained obese adults compared with the obese group following HIT. Indeed, anaerobic contributions to energy release increased significantly in the trained group following HIT. These higher anaerobic contributions were accompanied by both greater AOD deficits and larger decreases in oxygen uptake during SCE repetitions. Our results revealed that for the same relative work (at maximal power output), HIT may be an appropriate approach for enhancing anaerobic capacity, reasonable given that this type of exercise training improves glycolytic activity [25].

The performances demonstrated by our experimental subjects during SCE satisfied the criteria for supramaximal testing. Indeed, the PO exhibited by our groups during SCE was much closer to their maximal power output values (PO_max_), which were obtained during the force-velocity test (*p* = 0.1). Moreover, the PO exhibited by our groups did not differ during the fourth repetition. The AOD values obtained in our study were similar between the groups before HIT (74.4 vs. 73.4 ml.kg^−1^). Ten seconds of SCE may be considered a brief event during which subjects pedaled at maximal velocities against resistances determined during a force-velocity test [26, 27]. To meet the high ATP demand of SCE (e.g., adenosine diphosphate [ADP]), phosphorylation from PCr hydrolysis and glycolysis is necessary to overcome the oxygen deficit at the onset of exercise [28]. Several studies indicate that for events lasting 6–15 s, anaerobic contributions in sprinters are estimated to be 98 % of these individuals’ total energy expenditure [29]. Although AOD is a useful tool for determining anaerobic energy release [11], the present study demonstrated in the control group that the energy supply needed to perform 6 s during the first repetition of SCE results from anaerobic contributions (~83 %), as well as a small aerobic supply (~15 %). Therefore, contrary to the above findings, aerobic contributions to 6 s of SCE performance in obese adults represent a significant portion of the energy supply, which may result in lower rates of anaerobic glycolysis. The same tendency was also previously reported in normal-weight subjects, suggesting that this incompatibility between anaerobic energy release and power output during supramaximal exercise is sometimes compensated for by the increased contributions of aerobic metabolism, as reflected by the increase in oxygen consumption [26]. It is likely that the relative contributions of different energy systems during repeated-sprint exercises depend on the exercise protocol (duration, number of repetitions, recovery duration, passive or active recovery) and training status. In the context of obesity, we also speculate that other factors such as reduced motor-unit activation [8], premature fatigue and reduced activation of type II muscle fibers [7] may affect the energy contribution profile. Unfortunately, none of these factors were assessed in the present study.

During the second, third, and fourth repetitions, there were decreases in anaerobic contributions, which became significant during the third and fourth SCE bouts. Conversely, aerobic energy contributions increased and reached a value of participation of approximately 22 %. We speculate that examinations of repeated-sprint exercises may offer supplement information concerning changes in muscle metabolism and other factors affecting performance. In the present study, there was an increase in aerobic solicitation in both the control group and the trained group before HIT, during the second, the third, and the fourth repetitions. Given that our exercise was a model of intermittent exercise with 2 min of recovery between each repetition, it may be that the synthesis capacity of muscle was modified by obesity. In normal-weight subjects, several studies have indicated that the resynthesis of substrates such as PCr depends on several factors (endurance fitness, training status) [26, 30], factors that may affect the energy profile of muscle during subsequent exercise. Despite the absence of data regarding muscle recovery rates during supramaximal exercise in the obese population, the probable impact of obesity on the capacity of muscle resynthesis cannot be ruled out as an explanation for our results.

Following HIT, there was an 11 % reduction in calculated aerobic contributions to SCE, primarily as a result of an 11 % increase in anaerobic energy contributions. Moreover, the lactate response to the fourth SCE bout increased significantly following HIT. In the present study, the lactate concentrations were used to compare the anaerobic potential processes between subjects during this type of exercise [31]. Our results demonstrated that anaerobic capacity may improve after only 6 weeks of HIT, reflecting enhancements in glycolysis activation. Anaerobic capacity responses to HIT have typically been assessed by measuring blood lactate levels in response to standardized exercise load or by measuring anaerobic performance via a Wingate test. A number of studies have demonstrated that HIT lasting between 2 and 15 weeks results in significant increases in anaerobic capacity, increases ranging between 5 and 28 % in untrained males [21, 32, 33], as well as in overweight and obese men [10].

The mechanism(s) underlying these adaptations are likely the result of skeletal muscle adaptations involving marked increases in skeletal muscle capacity for glycolytic enzyme content [34], increases in anaerobic fitness, and increased insulin sensitivity. Additionally, it should be noted that obesity does not appear to negatively affect intrinsic muscle contractility properties (“muscle quality”) [35]. For Maffiuletti et al. [36], the presence of factors such as the handicapping effects of excess fat mass and impaired motor coordination may account for the poor physical performances of obese people of all ages. In the present study, a positive relationship between PO and both anaerobic contributions and lactate concentrations was noted. Concerning the lactate concentrations, which obtained via capillary blood samples using the LactatePro analyzer, higher values obtained after HIT may probably reflect amelioration in glycolysis activity in trained obese group. Although enhancements in anaerobic contributions were associated with PO enhancements, it is possible that the improvements in muscle coordination induced by HIT may also have contributed to PO and to lactate improvement.

From a clinical point of view, our results are valuable in understanding the interaction between intensity and energy contributions during high-intensity exercise. This study demonstrates that incompatibilities in energy balance during SCE bouts may reflect lower anaerobic contributions or poor skeletal muscle function in obese adults. This may, in turn, help clarify why obese people require high-intensity training as part of their multicomponent treatment for obesity.

The Wingate test (30 s of all-out sprint) has been the protocol utilized most often in the context of high-intensity training. This protocol consists of 3 to 4 min of cycle exercise per session, and each session typically occurs three times per week. This protocol, although remarkably short in duration, is extremely rigorous, and subjects must tolerate significant discomfort. Our study is the first to demonstrate that 6 weeks of very short repeated bouts of SCE may improve muscle power, improvements associated with increased anaerobic system contributions. In spite of these adaptations, more studies are necessary in order to understand the effects of such protocols on other health parameters. Finally, although the cardiovascular and metabolic consequences of obesity have been studied extensively over the last two decades, less attention has been paid to investigating the impact of obesity on muscle-energetic interactions for a given exercise. The present study has forced us to consider muscle energy potential during exercise in order to describe the true energetic adaptations that occur during exercise and following a training program for obese individuals.

However, there are some limits in the current study. Firstly, the missing post-intervention measure in several metabolic (e.g., lactate) and hormonal (e.g., catecholamines) parameters, besides psychological variables, may allow us to better understand the effect of such exercise training on metabolic and hormonal adaptations. Secondly, the physical activity and dietary level 48 h prior to the test were not directly controlled, and then we cannot be sure if our instructions were fully respected. Finally, the results obtained in the present study included men and women in the same study group. Given that gender could have affected our results, further studies are needed to address this effect.

## Conclusions

In conclusion, this study demonstrated the existence of a disparity in relative anaerobic and aerobic metabolism contributions to total energy release during supramaximal effort in obese adults, before and after HIT. Increased anaerobic contributions after HIT were associated with increased power output, although no changes in fat-free mass were observed. Such results highlight the importance of HIT in enhancing muscle tolerance to exercise by improving anaerobic capacity. Thus, the present findings provide an important first step towards an evidence-based approach for the utilization of HIT as a strategy for the obese sedentary population. However, the role of other training model (e.g., aerobic training) on improving several health parameters (e.g., cardiovascular and metabolic adaptations) cannot be excluded in the context of obesity management.

Finally, the evaluation of energy interactions during exercise must be examined in obese individuals.
